# Chemically Driven Ion Exchanging Synthesis of Na_5_YSi_4_O_12_-Based Glass-Ceramic Proton Conductors

**DOI:** 10.3390/ma16062155

**Published:** 2023-03-07

**Authors:** Toshinori Okura, Naoki Matsuoka, Yoshiko Takahashi, Naoya Yoshida, Kimihiro Yamashita

**Affiliations:** 1 Department of Applied Chemistry, School of Advanced Engineering, Kogakuin University, Nakano 2665-1, Hachioji, Tokyo 192-0015, Japan; 2 Graduate School of Medical and Dental Sciences, Tokyo Medical and Dental University, Yushima 1-5-45, Bunkyo, Tokyo 113-8501, Japan; 3 Division of Open Innovation, Advanced Comprehensive Research Organization, Teikyo University, Kaga 2-21-1, Itabashi, Tokyo 173-0003, Japan

**Keywords:** Na_5_YSi_4_O_12_, glass-ceramics, ion exchange, proton conductor

## Abstract

We have developed 12-membered silica-tetrahedra-ringed Na_5_YSi_4_O_12_-type sodium ion conducting glass-ceramics on the basis of the composition Na_3+3x-y_R_1-x_P_y_Si_3-y_O_9_ (R: rare earth elements; denoted as Narpsio); especially, the material of Na_4_Y_0.6_P_0.2_Si_2.8_O_9_ with the combined parameters of (x, y) = (0.4, 0.2) gives rise to the maximum conductivity of 1 × 10^−1^ Scm^−1^ at 300 °C. Because glass-ceramics generally have the advantage of structural rigidity and chemical durability over sintered polycrystalline ceramics, the present study employed glass-ceramic Narpsio to perform chemically driven ion exchange of Na^+^ with protonated water molecules with an aim to produce a proton conductor. The ion exchange was carried out in a hydrochloric acid solution by changing immersion time, temperature, and acid concentration. The ion exchanged Na_4_Y_0.6_P_0.2_Si_2.8_O_9_-based glass-ceramics were analyzed by the complex impedance method, and the proton conductivity was found to exhibit 3 × 10^−4^ Scm^−1^ at 300 °C with the activation energy of 59 kJ/mol. The dependence of humidity-sensitive conductivity of the ion exchanged bulk glass-ceramics was also examined; the conductivity increased almost linearly from 0.6 × 10^−4^ Scm^−1^ in dry air to 1.5 × 10^−4^ Scm^−1^ in 75% humid ambience at 300 °C. Thus, the ion exchanged glass-ceramics can be considered to be high temperature proton conductors as well as humidity sensors.

## 1. Introduction

Considering the exchanging feasibility of the charge-carriers with a different kind of ions in a solid electrolyte, some chemical methods have been studied for a long time in order to realize another kind of ion conductor. Using the first-generation ceramic sodium ion conductor of Na-β/β″-alumina (e.g., β: NaAl_11_O_17_ and β″: NaAl_5_O_8_), hydronium β/β″-alumina was obtained by acidic treatment of ceramic Na/K/-β/β″-alumina in order to avoid cracking during ion-exchange of Na^+^ and K^+^ ions with H_3_O^+^ in H_2_SO_4_ solution [1,2]. The second-generation conductor of NASICON (Na_1+x_Zr_2_P_3−x_Si_x_O_12_) was confirmed to thoroughly convert to proton conductors by ion exchange in acid, alkaline aqueous solution and even in water [3,4,5]. Currently NASICON-type Li_1+x_Ti_2_P_3-x_Si_x_O_12_ and Na_1+x_Ti_2_P_3-x_Si_x_O_12_ solid electrolytes were also demonstrated to perform ion exchange with hydronium ions [6,7]. In the third generation Na_5_RSi_4_O_12_ (R: rare-earth elements)-type sodium ion conductors [8,9], we employed the unprecedented field-assisted ion exchange method which applied dc voltage to a solid specimen at an elevated temperature of several tens in °C to release the induced strain during the process [10]. However, because of micro cracking during ion exchange of bulk specimens, no practically satisfactory bulk ion conductor has yet been produced by the ion exchange method for applications, such as solid electrolytes and sensors.

Our present aim is to produce bulk ceramic protonic conductors by ion exchange. Most of the abovementioned materials were polycrystalline ceramics or their powders. As glass-ceramics generally have structural rigidity against aqueous chemicals, we employed Na^+^ ion conductive glass-ceramics of Na_5_RSi_4_O_12_ (R: rare earth elements)-type sodium rare-earth silicophosphates in the system Na_2_O-R_2_O_3_-P_2_O_5_-SiO_2_, which will be abbreviated as Narpsio in the following [8,11]. We originally developed Na_5_YSi_4_O_12_-type Narpsio conductors on the basis of the composition Na_3+3x-y_Y_1-x_P_y_Si_3-y_O_9_ with appropriate parameters of x and y. Because Na^+^ ion glass-ceramics can be synthesized with the various combination of the composition parameters x and y, the use of Narpsio has an advantage to develop suitable materials for practical devices. 

The Na_5_YSi_4_O_12_ structure consists of tunneling channels of Na^+^ ions surrounded by 12-membered rings of SiO_4_ tetrahedra along the *c*-axis, forming conduction paths. A unit cell contains 14 mobile (15%) and 76 bonding (85%) Na^+^ ions. Narpsio materials were also found to be appropriate for the production of glass-ceramics without phase separation during crystallization from original glasses [11,12,13,14,15,16,17,18,19,20]. We currently found that those glass-ceramics can incorporate K^+^, two times larger than Na^+^ in volume, to some extent [21]. Considering the ion-exchanging capability, the present study focused on the fundamental glass-ceramic Narpsio of Na_4_Y_0.6_P_0.2_Si_2.8_O_9_ under (x, y) = (0.4, 0.2) which gives rise to the maximum conductivity of 1 × 10^−1^ Scm^−1^ at 300 °C [9,11,12,13,14] as the target material for conversion of sodium to hydronium ion conductors by chemical treatment in acid solutions. Narpsio glass-ceramics have another advantage of improved grain boundaries conduction due to the matrix-forming amorphous structure [11,12,13,14,15,16,17,18,19,20] because exuding Na^+^ ions in grains exchange with permeating H_3_O^+^ through grain boundaries. We firstly conducted ion exchange experiments using Narpsio powders to find the optimum conditions of the processing time, temperature, and solution concentration, then performed the production of protonic conducting bulk glass-ceramics under the appropriate conditions.

## 2. Materials and Measurements

### 2.1. Materials Preparation

**Figure 1:**
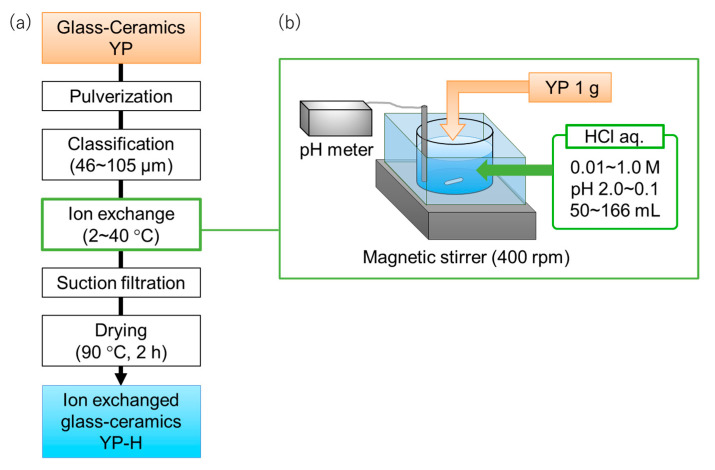
Flow diagram of materials preparation (**a**) and picture of ion exchange experiment (**b**).

The starting powders were synthesized from the reagents of anhydrous Na_2_CO_3_ (99.8% pure, Wako, Japan), Y_2_O_3_ (99.99% pure, Wako, Japan), NH_4_H_2_PO_4_ (99.0% pure, Wako, Japan), and SiO_2_ (99.9% pure, Wako, Japan). Those powders were weighed according to the composition of Na_4_Y_0.6_P_0.2_Si_2.8_O_9_, thereafter mixed by ball-milling. The mixture was calcined at the temperatures of 400 °C and 900 °C for 0.5 h to evacuate NH_3_ and CO_2_, respectively. The calcined powders were heated up to 1350 °C and melted in a Pt crucible for 1 h in air, then rapidly poured into cylindrical carbon dice to form glass. After annealing at 500 °C for 6h for nucleation, the annealed glass specimens were crystallized at 900 °C for 5 h according to the previous reports (Figure 1a) [11,12,13,14,15,16]. The relative densities of crystallized bulk specimens were evaluated by the Archimedes method in comparison with theoretical values, which were determined by pycnometry.

### 2.2. Ion Exchange Procedure

The abovementioned ion exchange of polycrystalline Na/K β/β″-Al_2_O_3_ was carried out by soaking in hot H_2_SO_4_. However, we employed dilute hydrochloric acid as the exchanging medium for gradual exchange. To evaluate the conditions for ion exchange, such as concentration of HCl aqueous solution and immersion period of solids, ball-milled and screened powders of glass-ceramics with a size of 46–105 μm in diameter were first subjected to ion exchange experiments (Figure 1a,b); the powders of 1 g were immersed in 50–166 mL HCl solution with various concentrations of 0.01 to 1.0 mol L^−1^ for 30 d (Figure 1a,b). The samples before and after ion exchange were named YP and YP-H. The ion exchange rate was determined by monitoring the pH of the solution (Equations (1)–(3)). The temperature effect of ion exchange was also evaluated according to the same manner mentioned above.

*r_ex_* = [H^+^]*_loss_*/[Na^+^]*_before-ex_* × 100 (1)

where *r_ex_*: ion exchange rare/%, [H^+^]*_loss_*: loss of [H^+^] in solution, and [Na^+^]*_before-ex_*: mobile [Na^+^] in sample before ion exchange.

[H^+^]*_loss_* = [H^+^]*_before__-ex_* − [H^+^]*_after__-ex_* = 10^−pH (*before-ex*)^ × *V_solution_* − 10^−pH (*after-ex*)^ × *V_solution_* (2)

where [H^+^]*_before-ex_*: [H^+^] in solution before ion exchange, [H^+^]*_after-ex_*: [H^+^] in solution after ion exchange, 10^−pH (*before-ex*)^: 10^−pH (*before ion exchange*)^/mol L^−1^, and *V_solution_*: volume of solution/L, 10^−pH (*after-ex*)^: 10^−pH (*after ion exchange*)^/mol L^−1^.

[Na^+^]*_before_*_-ex_ = *V_sample_* × [Na^+^]*_in 1g_* (3)

where *V_sample_*: volume of sample/g, and [Na^+^]*_in 1g_*: mobile [Na^+^] in 1 g of sample /mol g^−1^.

### 2.3. XRD, SEM, and Thermal Analyses of Glass and Glass-Ceramics

Identification of the crystalline phases was performed on the crushed powders of crystallized specimens by the X-ray diffraction (XRD; Rigaku MiniFlex II) method. The XRD measurements were calculated between 10 and 40° under the scanning speed of 3°·min^−1^. Scanning electron micrographic (SEM; JEOL JSM-6701F) observation was also performed on the bulk samples chemically etched with 3% HF solution to evaluate the microstructure. The conductive coatings were performed using sputtering of carbon. Differential scanning calorimetry (DSC) was carried out on the prepared samples to confirm the changes in thermodynamic parameters before and after ion exchange. The sample was packed in an aluminum pan, covered with an aluminum lid, and measured using a Rigaku DSC_8230. Thermogravimetric-mass spectrometry (TG-MS) was performed to analyze the gaseous components desorbed from the sample. The sample was packed in an alumina pan and measured simultaneously using a thermogravimetric (Netzsch, TG209 F1 Libra)-mass spectrometer (Netzsch, QMS403 D Aëolos) under the conditions of the heating rate of 10 °C min^−1^, the He gas flow rate of 20 mL min^−1^, and the sampling span of 10 s.

### 2.4. Measurements of Conduction Properties

Electrochemical impedance measurements were undertaken by the alternate current (AC) two-probe method on cylindrical glass-ceramics with a diameter and thickness of 14 mm and 2 mm, respectively. Electrodes were formed by sputtering gold on the polished surfaces. The ionic conductivities were measured by impedance spectroscopy (Solartron Analytical 1260A Impedance Analyzer and 1296A Dielectric Interface system). The applied AC field was in the frequency range from 10 mHz to 32 MHz, with a voltage amplitude of 300 mV. The temperature dependence of the conductivity was measured similarly at several temperatures ranging from 100 °C to 300 °C.

**Figure 2:**
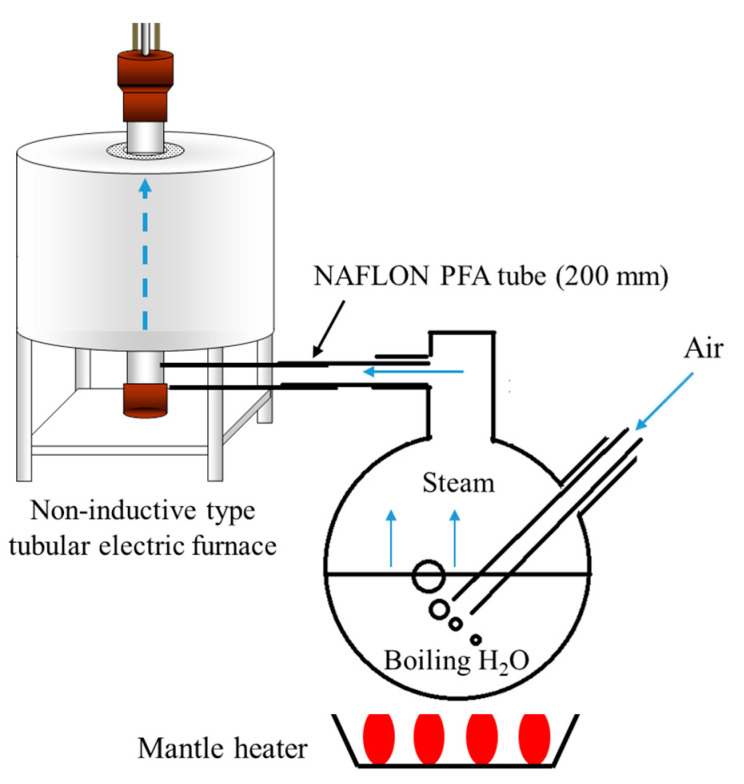
Apparatus for conductivity measurements under steam stream.

Furthermore, the conductivity of H^+^ conductive glass-ceramics was measured under water vapor atmosphere to evaluate the effect of relative humidity on H^+^ conductivity. The schematic diagram of the measurement apparatus is shown in Figure 2. Water in an eggplant flask was heated with a mantle heater to generate steam. The water was bubbled using an air pump, and the generated steam was introduced into a non-inductive tubular electric furnace. By changing the temperature of the mantle heater, the relative humidity of the water vapor on the introduction side was changed, and the conductivity was measured. The relative humidity was calculated by Equation (4).

*φ* = [*e*/*e_s_* (*t*)] × 100 (4)

where *φ*: relative humidity/%, *e*: partial pressure of water vapor/hPa, *t*: temperature/°C, and *e_s_* (*t*): saturated water vapor pressure/hPa. To calculate the saturated water vapor pressure, the Tetens equation shown in Equation (5) was used.

*e_s_* (*t*) = 6.1078 × 10^*at*/(*b* + *t*)^ (5)

where *a* = 7.5, *b* = 237.3 (for water), *a* = 9.5, and *b* = 265.5 (for ice).

In order to calculate the amount of water vapor depending on the temperature of the mantle heater, the dew point of the generated water vapor was measured using the low-pressure cooling dew-point method. Figure 2 shows the macroscopic dew-point meter used. After the temperature of the mantle heater reached the set temperature, air was introduced from the pump to bubble the deionized water in the eggplant flask. The temperature inside the apparatus and the temperature of the generated steam were kept at the same level by holding the flask in this state for a sufficient period of time. The glass tube, which is the outlet port, was then immersed in water maintained at a temperature of 95 °C or higher, and the temperature of the water was gradually lowered. The temperature at which dew condensed inside the glass tube was taken as the dew point of the generated water vapor.

The ionic conductivities in a steam atmosphere were measured using an Agilent Technologies 4294A-Precision-Impedance Analyzer, similar to the method described above. The applied AC field had a frequency range from 40 Hz to 100 MHz, with a voltage amplitude of 300 mV. The temperature dependence of the conductivity was measured similarly at several temperatures ranging from 100 °C to 300 °C.

## 3. Results and Discussion

### 3.1. Characterization of Original Narpsio Glass-Ceramics

**Figure 3:**
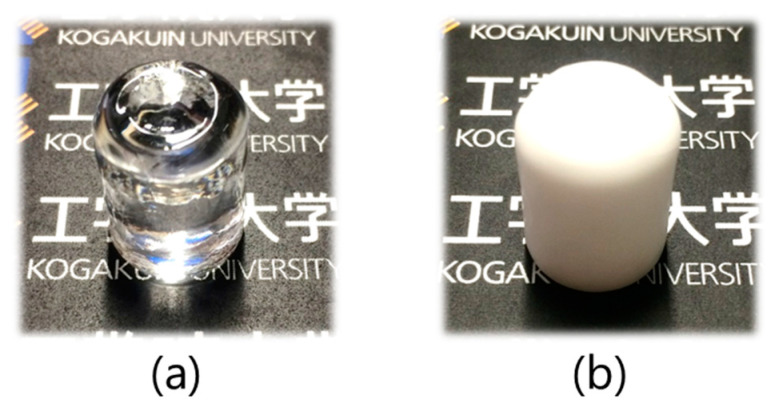
Photographs of original glass (**a**) and crystallized glass-ceramics (**b**) of Narpsio.

The original glass and crystallized glass-ceramic specimens are compared to demonstrate the dense bodies in Figure 3. As can be seen in the figure, the precursor bulk glasses were transparent without apparent pores. The crystallized glass-ceramics were more than 98% dense of the theoretical value, and the crystalline phase of glass-ceramic specimens was confirmed as Na_5_RSi_4_O_12_-type by XRD by comparison with the international standard diffraction pattern (ICDD No. 00-032-1204 for Na_5_YSi_4_O_12_).

### 3.2. Ion Exchange of Narpsio Glass-Ceramics

**Figure 4:**
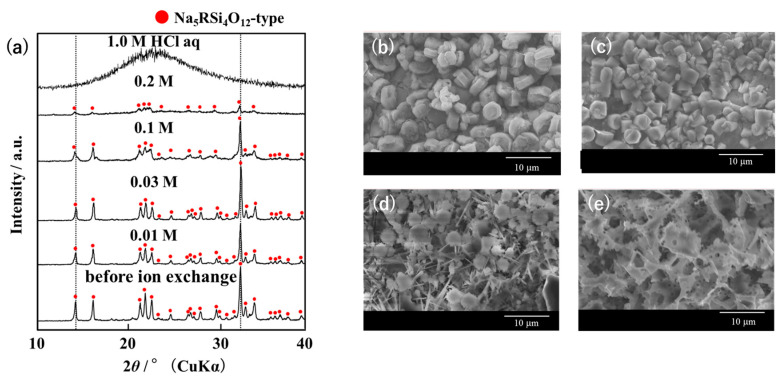
XRD patterns (**a**); SEM photographs of starting (**b**); ion exchanged Narpsio glass-ceramics in 0.03 M (**c**); 0.1 M (**d**); 1.0 M HCl aqueous solutions (**e**). The red solid circles in Figure 4a denote the peaks assigned to the Na_5_RSi_4_O_12_-type structure, which are abbreviated as N5 for convenience.

XRD analysis showed that when Narpsio powders were immersed in an aqueous HCl solution for 30 d at room temperature, the original Na_5_RSi_4_O_12_-type structure was maintained in the HCl concentration range of 0.01 to 0.1 M, whereas the crystalline phase was destroyed to amorphous over 0.2 to 1.0 M (Figure 4a). The SEM observation supported the preservation of the original structure in the dilute HCl concentration, while 1.0 M concentrated HCl dissolved the glass-ceramic surface (Figure 4b–d).

**Figure 5:**
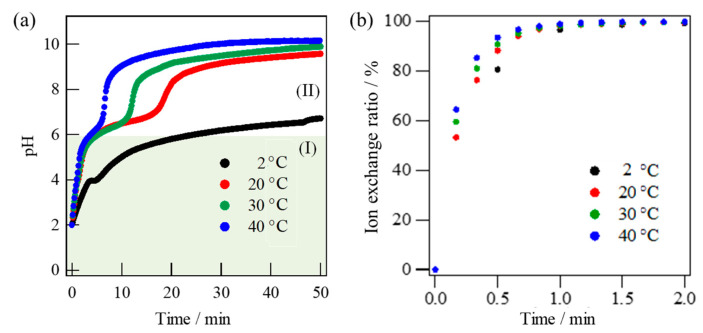
Time-changes of pH values (**a**) and Na^+^ exchanged rates (**b**) during ion exchange of Narpsio glass-ceramic powders at various temperatures.

The monitored pH change of the solution with immersion time indicates that the ion exchange proceeded more rapidly from pH = 2 to an alkaline region up to pH = 10 via a two-step processes at a higher temperature without structural destruction (Figure 5a). Based on the result of Figure 5a, the ion exchange ratio was calculated using Equation (1). The evaluated result is shown in Figure 5b. It was also noted that the ion exchange was completed within a few minutes at the first step I, even at a low temperature of 2 °C (Figure 5b). Possibly the second step II was considered to give rise to a slight surface corrosion, because the surface morphology was seen to be roughened by scattered pores (Figure 4d,e).

**Figure 6:**
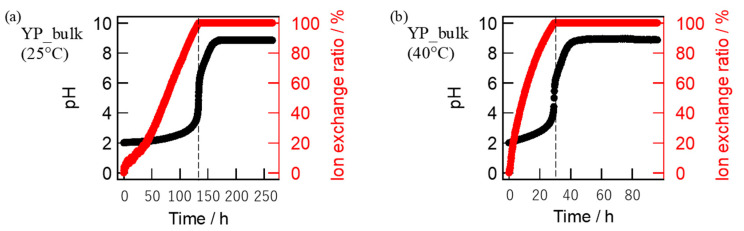
Time changes of pH values and Na^+^ exchanged rates during ion exchange of Narpsio glass-ceramic bulks at the temperatures of 25 °C (**a**) and 40 °C (**b**).

The ion exchange on bulk glass-ceramics took place much more slowly according to the two steps; the first step to exchange mobile Na^+^ ions of the bulk specimens with oxonium ions in solutions was accomplished for 130 h at 25 °C (Figure 6a) and 30 h at 40 °C (Figure 6b).

**Figure 7:**
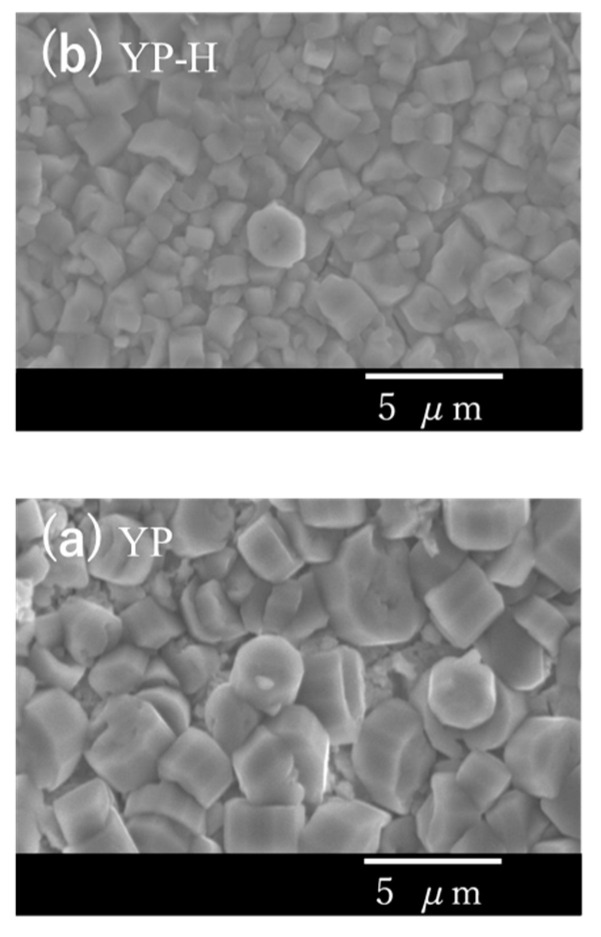
SEM photographs of Narpsio glass-ceramic surfaces before (**a**) and after (**b**) ion exchange at 25 °C using 0.01 M HCl aqueous solution.

**Figure 8:**
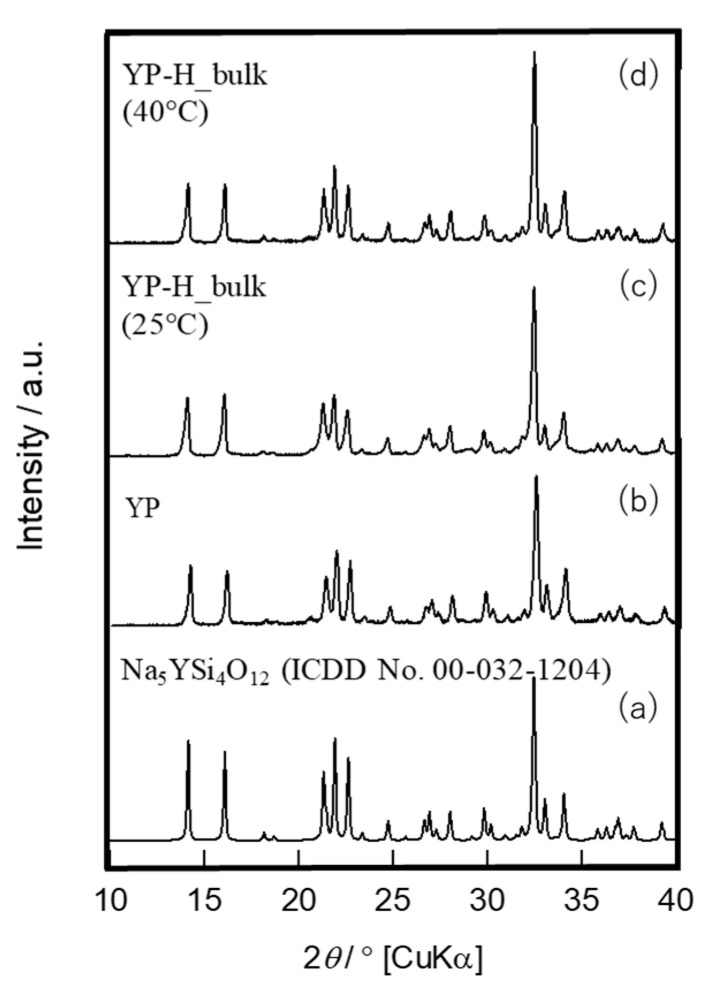
XRD patterns of ICDD data (**a**); original (**b**); ion exchanged bulk exchange at 25 °C (**c**); and 40 °C (**d**).

The surfaces of bulk glass-ceramic specimens thus treated exhibited no considerable change after ion exchange by SEM observation (Figure 7a,b), and XRD analysis confirmed the no destruction of the Na_5_RSi_4_O_12_-type structure (Figure 8a–d). However, as the longer time treatment at 25 °C gave mechanical damage to specimens, the temperature of 60 °C was chosen for the preparation of hydronium bulk glass-ceramics.

**Figure 9:**
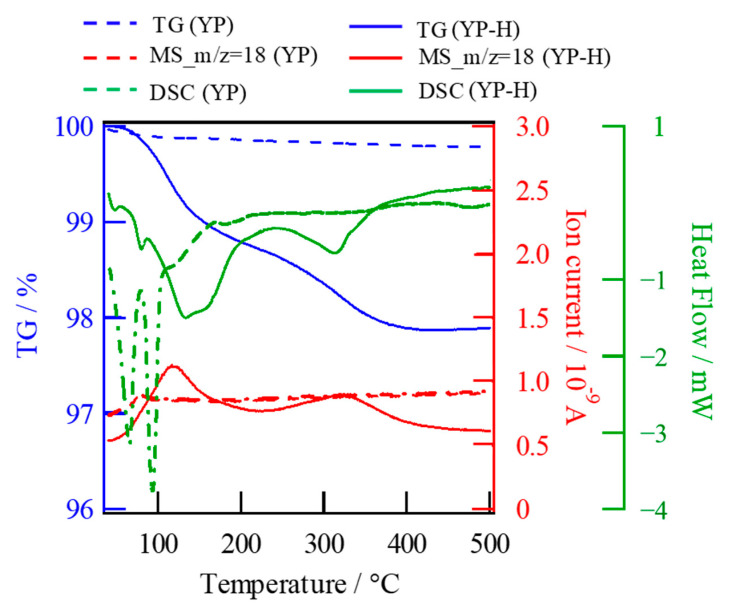
TG-MS and DSC curves measured on Narpsio bulk glass-ceramics before and after ion exchange.

Endothermic peaks on HCl-untreated specimens were observed at 64.1 and 91.9 °C, and the total weight loss was within 2%, while the peaks were detected at 133.3 and 312.6 °C in addition to 47.6 and 79.6 °C on ion-exchanged ones (Figure 9). All peaks observed below 100 °C were attributed to evaporation of adsorbed water on surfaces. However, the endothermic peaks at 133.3 and 312.6 °C on ion-exchanged specimens are deduced to the evaporation of ion exchanged hydrated water from the inner bulks. The amount of water loss as protons from the bulk specimen was evaluated as 0.794 mol per 1 mol of glass-ceramics by TG analysis, while the mobile Na^+^ concentration was calculated as 0.622 mol per 1 mol of glass-ceramics by comparison of specimen’s weight with chemical formula. The consistency of the two values with each other within the experimental error indicates the one-to-one exchange of Na^+^ of a glass-ceramic with H^+^ of HCl solution.

### 3.3. Conduction Properties of Ion Exchanged Narpsio Glass-Ceramics

**Figure 10:**
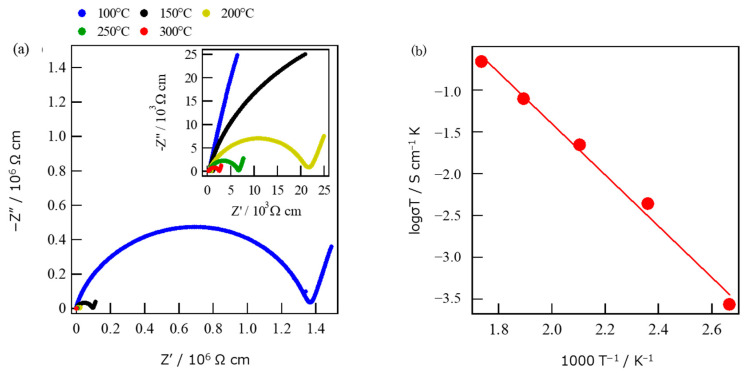
Cole–Cole (**a**) and Arrhenius (**b**) plots measured on ion exchanged bulk glass-ceramics.

The measured complex impedances are shown as an Arrhenius plot, where a single semicircle was observed at each temperature (Figure 10a,b). The result indicates that the separation of grains and grain boundaries-conductions were inseparable, and then the measured conductivities (σ) corresponded to the total of the two microstructural elements. The value of σ at 300 °C was 3.8 × 10^−4^ Scm^−1^ with the activation energy of 58.6 kJ mol^−1^ for the most conductive bulk specimen. There are two main types of proton transfer: Grotthuss type and Vehicle type. In the Grotthuss type, a proton becomes H_3_O^+^ when it receives an electron supply from an unpaired electron in the presence of water and can move by hopping against an unpaired electron on a neighboring water molecule when there are enough neighboring water molecules. In this case, the proton conduction can be achieved with a low activation energy because it does not require much energy for translational motion. On the other hand, in the Vehicle type, protons are given unpaired electrons by water molecules to become H_3_O^+^, and then they form a group and perform translational motion to conduct protons. A large amount of activation energy is required for the translational motion [22]. The proton transfer in crystallized glass after carrier ion exchange is considered to be of the Vehicle type due to the high activation energy. Although the conductivity of glass-ceramics decreased in the order of 10^−2^ Scm^−1^ by ion exchange of Na^+^ with H_3_O^+^, the value of σ of ion exchanged glass-ceramics can still be anticipated to be on the level of practical applications with further efforts. Because the microstructure of glass-ceramics strongly depends upon annealing time and temperature of nucleation and crystal growth, we are investigating the effects of those parameters on improvement of conduction properties.

**Figure 11:**
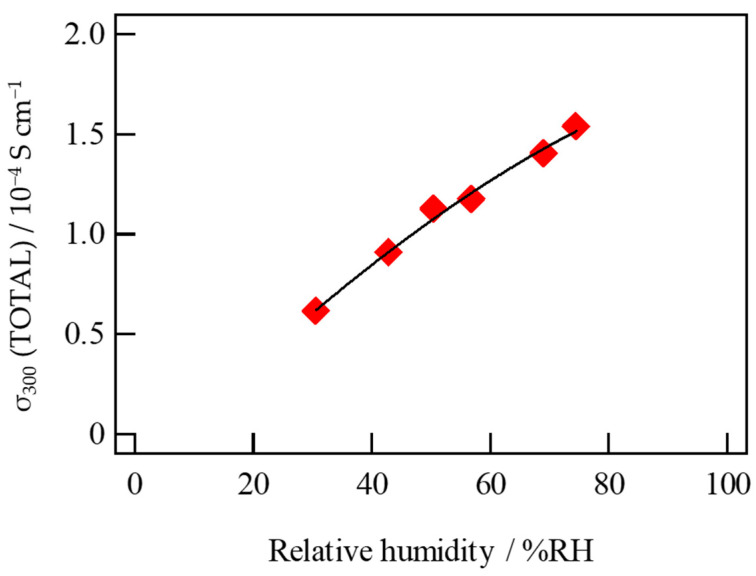
Humidity-sensitive conductivity measured on ion exchanged bulk glass-ceramics at 300 °C.

Although the top value of σ of ion exchanged bulk specimens reached to ca. 3 × 10^−4^ Scm^−1^ at 300 °C, the conductivities were usually a little scattered. This can be attributed to the difference in unstable concentration of incorporated protons among specimens. With the aim at clarification of water vapor effect on conductivity, we conducted the σ measurements under various controlled humidity at 300 °C. The value of σ increased from 0.6 × 10^−4^ Scm^−1^ in dry air to 1.5 × 10^−4^ Scm^−1^ under 75% humidity, indicating humidity-sensitive conduction in bulk specimens (Figure 11). As the conductivity of the bulk specimen varies almost linearly with humidity, the ion exchanged glass-ceramics can be applicable to humidity sensors at an elevated temperature, such as 300 °C. As for the water resistance of the samples, there is concern about the possibility of elution of bonding Na^+^ during charge carrier ion exchange or under high-humidity conditions, so it is required in the future to improve the water resistance by adding Al or Zr.

## 4. Conclusions

The present study focused on the fundamental Na_5_YSi_4_O_12_-type glass-ceramic Narpsio on the basis of the composition Na_3+3x-y_Y_1-x_P_y_Si_3-y_O_9_, of which Na_4_Y_0.6_P_0.2_Si_2.8_O_9_ with the combined parameters of (x, y) = (0.4, 0.2) as the target material for conversion of sodium to hydronium ion conductors by chemical treatment in acid solutions. Taking into account the structural rigidity and chemical durability of glass-ceramics, chemically driven ion exchange of Na^+^ with protonated water molecules in Na_4_Y_0.6_P_0.2_Si_2.8_O_9_-based glass-ceramics was performed. The value of σ at 300 °C was 3.8 × 10^−4^ Scm^−1^ with the activation energy of 58.6 kJ mol^−1^ for the most conductive bulk specimen. The ion exchanged bulk glass-ceramics showed humidity-sensitive conductivity; σ increased from 0.6 × 10^−4^ Scm^−1^ in dry air to 1.5 × 10^−4^ Scm^−1^ in 75% humid ambience at 300 °C. Although the conductivity of glass-ceramics decreased in the order of 10^−2^ Scm^−1^ by ion exchange of Na^+^ with H_3_O^+^, the value of σ of ion exchanged glass-ceramics still can be anticipated to be on the level of practical applications with further efforts. The ion exchange is a promising method to convert the charge carrying ions to the different ones because the finding of a novel conductor is unimaginably beyond our effort. The use of glass-ceramics is also noticeable because the fabrication of thinner bulk specimens can be realized through the glass making process, where melts are thinned during cooling. Considering those circumstances, we are conducting the research to optimize ion exchange conditions for glass-ceramic materials to improve the proton conductivity up to ca. 10^−2^ Scm^−1^ for practical applications.

## Data Availability

The data are available on request.
